# Quinine, an old anti-malarial drug in a modern world: role in the treatment of malaria

**DOI:** 10.1186/1475-2875-10-144

**Published:** 2011-05-24

**Authors:** Jane Achan, Ambrose O Talisuna, Annette Erhart, Adoke Yeka, James K Tibenderana, Frederick N Baliraine, Philip J Rosenthal, Umberto D'Alessandro

**Affiliations:** 1 Department of Pediatrics and Child Health, Makerere University College of Health Sciences, P.O. Box 7475, Kampala, Uganda; 2 Department of Epidemiology and Biostatistics, Makerere University School of Public Health, P.O Box 7072, Kampala, Uganda; 3 Department of Parasitology, Institute of Tropical Medicine, Nationalestraat 155, 2000 Antwerp, Belgium; 4 Epidemiology Unit, Uganda Malaria Surveillance Project, P.O Box 7475, Kampala, Uganda; 5 Communicable Diseases Control Department, Malaria Consortium Africa, P.O Box 8045, Kampala, Uganda; 6 Department of Medicine, University of California San Francisco, 1001 Potrero Ave, SFGH 30, San Francisco, CA, 94143, USA

## Abstract

Quinine remains an important anti-malarial drug almost 400 years after its effectiveness was first documented. However, its continued use is challenged by its poor tolerability, poor compliance with complex dosing regimens, and the availability of more efficacious anti-malarial drugs. This article reviews the historical role of quinine, considers its current usage and provides insight into its appropriate future use in the treatment of malaria. In light of recent research findings intravenous artesunate should be the first-line drug for severe malaria, with quinine as an alternative. The role of rectal quinine as pre-referral treatment for severe malaria has not been fully explored, but it remains a promising intervention. In pregnancy, quinine continues to play a critical role in the management of malaria, especially in the first trimester, and it will remain a mainstay of treatment until safer alternatives become available. For uncomplicated malaria, artemisinin-based combination therapy (ACT) offers a better option than quinine though the difficulty of maintaining a steady supply of ACT in resource-limited settings renders the rapid withdrawal of quinine for uncomplicated malaria cases risky. The best approach would be to identify solutions to ACT stock-outs, maintain quinine in case of ACT stock-outs, and evaluate strategies for improving quinine treatment outcomes by combining it with antibiotics. In HIV and TB infected populations, concerns about potential interactions between quinine and antiretroviral and anti-tuberculosis drugs exist, and these will need further research and pharmacovigilance.

## Background and historical perspective

The discovery of quinine is considered the most serendipitous medical discovery of the 17th century [1] and malaria treatment with quinine marked the first successful use of a chemical compound to treat an infectious disease[2]. Quinine, as a component of the bark of the cinchona (quina-quina) tree, was used to treat malaria from as early as the 1600s, when it was referred to as the "Jesuits' bark," "cardinal's bark," or "sacred bark." These names stem from its use in 1630 by Jesuit missionaries in South America, though a legend suggests earlier use by the native population[2]. According to this legend, an Indian with a high fever was lost in an Andean jungle. Thirsty, he drank from a pool of stagnant water and found that it tasted bitter. Realizing that the water had been contaminated by the surrounding quina-quina trees he thought he was poisoned. Surprisingly, his fever soon abated, and he shared this accidental discovery with fellow villagers, who thereafter used extracts from the quina-quina bark to treat fever [3]. The legend of quinine's discovery accepted in Europe differs though, and involves the Spanish Countess of Chinchon who, while in Peru, contracted a fever that was cured by the bark of a tree. Returning to Spain with the bark, she introduced quinine to Europe in 1638 and, in 1742, botanist Carl Linnaeus called the tree "Cinchona" in her honour [4].

Before 1820, the bark of the cinchona tree was first dried, ground to a fine powder, and then mixed into a liquid (commonly wine) before being drunk. In 1820, quinine was extracted from the bark, isolated and named by Pierre Joseph Pelletier and Joseph Caventou. Purified quinine then replaced the bark as the standard treatment for malaria [5]. Quinine and other cinchona alkaloids including quinidine, cinchonine and cinchonidine are all effective against malaria. The efficacies of these four alkaloids were evaluated in one of the earliest clinical trials, conducted from 1866 to 1868 in 3600 patients using prepared sulfates of the alkaloids. With the main outcome measure of "cessation of febrile paroxysms", all four alkaloids were found to be comparable, with cure rates of >98%[6]. However, after 1890 quinine became the predominantly used alkaloid, mainly due to a change in supply from South American to Javan cinchona bark, which contained a higher proportion of quinine [7]. Quinine remained the mainstay of malaria treatment until the 1920s, when more effective synthetic anti-malarials became available. The most important of these drugs was chloroquine, which was extensively used, especially beginning in the 1940s [6]. With heavy use, chloroquine resistance developed slowly. Resistance of *Plasmodium falciparum *to chloroquine was seen in parts of Southeast Asia and South America by the late 1950s, and was widespread in almost all areas with falciparum malaria by the 1980s. With increasing resistance to chloroquine, quinine again played a key role, particularly in the treatment of severe malaria [6]. To-date quinine continues to play a significant role in the management of malaria. This review, discusses the historical role of quinine, considers its current usage, and provides insight into the appropriate future use of quinine for the treatment of malaria. Information was obtained by searching published literature in the National Library of Medicine via Pub Med and MEDLINE search engines for research articles, reviews, books, and other reports. Identification of published reports was done using key word searches such as quinine and malaria treatment, quinine and drug resistance, quinine in pregnancy, quinine and antibiotic combinations, and quinine and HIV/TB infected populations.

## Quinine properties

Quinine is a cinchona alkaloid that belongs to the aryl amino alcohol group of drugs. It is an extremely basic compound and is, therefore, always presented as a salt[6]. Various preparations exist, including the hydrochloride, dihydrochloride, sulphate, bisulphate, and gluconate salts; of these the dihydrochloride is the most widely used. Quinine has rapid schizonticidal action against intra-erythrocytic malaria parasites. It is also gametocytocidal for *Plasmodium vivax *and *Plasmodium malariae*, but not for *Plasmodium falciparum*. Quinine also has analgesic, but not antipyretic properties. The anti-malarial mechanism of action of quinine is unknown.

Quinine is rapidly absorbed both orally and parenterally, reaching peak concentrations within 1-3 hours[8]. It is distributed throughout the body fluids and is highly protein bound, mainly to alpha-1 acid glycoprotein. The binding capacity in plasma is concentration dependent, but also depends on the levels of alpha-1 acid glycoprotein, which therefore makes comparisons between different studies difficult[9]. Quinine readily crosses the placental barrier and is also found in cerebral spinal fluid. Excretion is rapid - 80% of the administered drug is eliminated by hepatic biotransformation and the remaining 20% is excreted unchanged by the kidney [10-12]. The half-life of quinine ranges between 11-18 hours [13,14]. Several pharmacokinetic characteristics of quinine differ according to the age of the subject and are also affected by malaria. The volume of distribution is less in young children than in adults, and the rate of elimination is slower in the elderly than in young adults. In patients with acute malaria the volume of distribution is reduced and systemic clearance is slower than in healthy subjects; these changes are proportional to the severity of the disease. As a result, plasma quinine levels are higher in patients with malaria. Protein binding of quinine is increased in patients with malaria as a result of an increased circulating concentration of alpha-1 acid glycoprotein [15].

Quinine has a low therapeutic index, and adverse effects with its use are substantial [16]. The side effects commonly seen at therapeutic concentrations are referred to as cinchonism, with mild forms including tinnitus, slight impairment of hearing, headache and nausea. Impairment of hearing is usually concentration dependent and reversible [17]. More severe manifestations include vertigo, vomiting, abdominal pain, diarrhea, marked auditory loss, and visual symptoms, including loss of vision. Hypotension may occur if the drug is given too rapidly, and venous thrombosis may occur following intravenous injections [10]. Intramuscular administration is painful and may cause sterile abscesses. Hypoglycaemia is yet another common side effect of quinine therapy [15,18] and is a particular problem in pregnant women[19]. Hypoglycaemia has been reported to occur in up to 32% of patients receiving quinine therapy[18]. However in more recent studies, hypoglycaemia occurred in only 3% of adults and 2.8% of African children receiving quinine [20,21]. Less frequent but more serious side effects of quinine therapy include skin eruptions, asthma, thrombocytopaenia, hepatic injury and psychosis [22].

## Overview of quinine use in the management of malaria

Quinine remains an important anti-malarial drug, almost 400 years after Jesuit priests first documented its effectiveness. The 2010 World Health Organisation (WHO) guidelines recommend a combination of quinine plus doxycycline, tetracycline or clindamycin as second-line treatment for uncomplicated malaria (to be used when the first-line drug fails or is not available) and quinine plus clindamycin for treatment of malaria in the first trimester of pregnancy [23]. Based on recent trials, intravenous artesunate should be used for the treatment of severe falciparum malaria in adults [20] and children[21], in preference to quinine.

By 2009, 31 African countries recommended quinine as second-line treatment for uncomplicated malaria, 38 as first-line treatment of severe malaria and 32 for treatment of malaria in the first trimester of pregnancy [24]. In most of Africa, quinine is still used as monotherapy, contrary to the WHO recommendations[23,24]; the reason for this practice may be the higher costs of quinine-antibiotic combinations. Quinine continues to play a significant role in the management of malaria in sub-Saharan Africa and other malaria endemic areas, and its use in routine practice may not be restricted to the stated WHO recommendations. In Cameroon, even one year after the introduction of ACT, quinine continued to be used as first-line therapy, with 45% of adults receiving oral quinine for uncomplicated malaria [25]. Recent surveillance data from sentinel sites in Uganda showed that quinine was prescribed for up to 90% of children < 5 years with uncomplicated malaria [26].

The use of quinine for uncomplicated malaria cases should have decreased due to toxicities, poor compliance and the implementation of newer and better tolerated therapies such as ACT. However, the limited availability of ACT and the increasing resistance to chloroquine and antifolates have actually increased its use in recent times [27]. Therefore, studies evaluating the role of quinine in the management of malaria have been reviewed.

## Quinine for uncomplicated malaria

In several settings, oral quinine continues to be used as treatment for uncomplicated malaria, a practice mainly resulting from frequent stock-outs of the recommended ACT [26,28]. Previous studies of the effectiveness and efficacy of quinine for uncomplicated malaria showed mixed results (Table 1). The majority of these studies were conducted in settings with reported declining efficacy of quinine in Southeast Asia and South America. Earlier studies in these regions, using varying dosing regimens, showed cure rates ranging from 76% to 98%. The lower cure rates were mainly observed with shorter regimens (3 days) and higher cure rates when the drug was combined with sulphadoxine-pyrimethamine, tetracycline or clindamycin [29-34]. Similar findings were reported in Vietnam, where a three-day course of quinine plus artesunate had a cure rate of only 50%, compared to a five-day course, which had a cure rate of 76%[35]. Studies in Southeast Asia using quinine monotherapy for 7 days showed cure rates of 85-87% [29,33], which is similar to what was observed over 15 years earlier [36], (Table 1).

**Table 1:** Summary of studies of quinine for the treatment of uncomplicated malaria

Study site	Year	Sample size and study population	Drug Regimens	Duration of follow-up	Treatment outcome	Comment	Reference
Thailand, region with multidrug resistant malaria	1984-1985	66 children 2-12 years	Quinine Quinidine	28 days DOT	Cure rates: Quinine - 85% Quinidine - 88%	Treatment failures only RI responses	[29]

Cambodia, region with multidrug resistant malaria	1983	119 adults, >15 years	Mefloquine +SP (MSP) 3 days quinine+tetracycline (Q3T7) 7 days of quinine+ tetracycline (Q7T7)	28 days DOT	Cure rates: MSP: 98% Q3T7: 76% Q7T7: 92%	Q7T7 still gives good cure rate	[30]

Brazil, setting with quinine resistance	1985	100 patients 18-55 years	Mefloquine 1000 mg single dose (MQ) 3 days quinine+SP (Q3+SP)	42 days DOT	Cure rates: MQ: 96% Q3 + SP: 98%	Four RI responses in Q3 + SP group	[31]

Thailand, region with multidrug resistant malaria	1994	102 patients 16-60 years	Mefloquine+tetracycline (MQT) 7 days of Quinine+ tetracycline (Q7T7)	28 days DOT	Cure rates: MQT: 94% Q7T7: 98%	MQ + Tetra as effective as Q7T7	[34]

Thailand, region with multidrug resistant malaria	1995-1997	204 male patients 15-64 years	7 days quinine (Q7) Quinine + tetracycline (Q7T7) Quinine + clindamycin (Q7C7)	28 days Directly observed therapy	Cure rates: Q7: 87% Q7T7: 98% Q7C7: 100%	Tetracycline or clindamycin improves quinine cure rates	[33]

Equatorial Guinea, setting with no quinine resistance	1999	114 children 6-59 months	7days quinine (Q7) Chloroquine (CQ) Sulfadoxine/pyrimethamine (SP)	114 day follow-up	Cure rates: Q7: 94.5% CQ: 60% SP: 90%	Quinine is effective against P.falciparum malaria	[43]

Cameroon, High transmission setting	2005	30 children 0.5-6 years	5 days quinine (Q5)	14 day follow-up	Cure rates: 100%		[41]

Burundi Perennial transmission setting	1992-1995	472 children 0-14 years	Chloroquine (CQ) 5 days quinine (Q5)	7 day follow-up	Failure rates Q5: 1992-1993: 4.2% 1994-1995: 7.1%		[40]

Guinea-Bissau Perennial transmission setting	1994-1995	203 children 0.7-13 years	3 days quinine (Q3) 5 days quinine (Q5) 7 days quinine (Q7)	28-35 day follow-up	Day 28 recurrent parasitemia: Q3: 79% Q5: 90% Q7: 11%	3 day quinine regimens should not be used.	[37]

Gabon High transmission setting	1993-1994	120 adults = 15years	3 days quinine (Q3) 3 days quinine+clindamycin (Q3C3) 3 days quinine+doxycycline (Q3D3)	28 day follow-up	Day 28 cure rates: Q3: 38% Q3C3: 92% Q3D3: 91%	The two short course combinations of quinine had excellent cure rates	[109]

Uganda Meso-endemic transmission setting	2007-2008	175 children 6months - 5 years	7 days quinine (Q7) 3 days artemether-lumefantrine (AL)	28 day follow-up	Cure rates: Q7: 64% AL: 97%	Results question the advisability of quinine use for uncomplicated malaria	[45]

Moreover, the addition of either tetracycline or clindamycin to quinine in the Thai study improved cure rates to 98% and 100% respectively and also delayed the appearance of *Plasmodium vivax *infection, suggesting additional activity against this species [33].

In Africa, studies evaluating three-day quinine treatment regimens have usually found unacceptably high failure rates [37], with recurrent infections at day 28 post-treatment experienced in 30% - 50% of patients[37-39]. However most of these studies did not perform PCR analyses to distinguish between recrudescence and re-infection, leading to possible underestimation of efficacy. In interpreting these results, the malaria transmission intensity at the study sites needs to be taken into consideration, as high treatment failure rates in high transmission settings may be due to a high risk of new infections. Additional PCR unadjusted studies that have evaluated five-day regimens of quinine have found recurrent infection rates on day 7 between 4% and 7% [40] and day 14 treatment failure rates of 0 to 5% (Table 1) [41,42]. In Equatorial Guinea, five-day courses of quinine were associated with day 14 PCR unadjusted failure rates as high as 22%. These latter results prompted a change in the quinine treatment regimen for this region to a 7 day course, with subsequent significant decrease in treatment failure rates to 3%-5.5% [43]. This study also reported that treatment failure rates with quinine remained stable over the five-year period of surveillance.

Even with seven-day treatment durations, evaluations of different quinine dosage regimens have revealed interesting trends. Doses of 10 mg/kg/day given twice daily for 7 days were associated with day 28 treatment failure rates as high as 30%[37]. Increasing the quinine dosage to 15 mg/kg/day or 20 mg/kg/day improved treatment outcomes, with failure rates ranging from 8% to 14%[37], although potential increases in toxicity with higher dosages are a concern. The treatment regimen currently recommended in sub-Saharan Africa is 10 mg/kg of the base given 8 hourly for 7 days. This regimen was associated with a lower rate of recurrent infections on day 28 (6.3%) compared to the 10 mg/kg twice daily regimen (16.1%)[44].

The advent of ACT has provided important new therapeutic options for the management of uncomplicated malaria in regions with high prevalence of multi-drug resistant malaria. A few available trials have shown superiority of ACT over quinine in the management of uncomplicated malaria [32,45,46]. In Brazil, patients treated with artemether-lumefantrine (AL) had significantly faster parasite clearance times when compared to those treated with quinine+doxycycline [46]. Considering the extensive available data, quinine should not be used to treat uncomplicated malaria when ACT is available [27,45]. ACT has the advantages of simplicity of dosing, which promotes adherence to therapy when compared with the seven-day treatment courses of quinine [32,45], better tolerance and decreased risks of serious toxicity.

Nevertheless, despite their scale up in Africa, the cost and availability of ACT in the public sector remains a major challenge. In 2008, ACT coverage in the public sector in high-burden African countries was only 42%[47]. Similarly, a survey carried out during the same year in seven African countries showed that the percentage of fever cases in children < 5 years treated with ACT was only 16% [47]. The sustainability of ACT supplies in resource limited settings therefore presents a huge problem, with stock-outs consistently occurring in health facilities [48]. Quinine, on the other hand, is a relatively cheap drug and often the only available option, rendering its rapid withdrawal for uncomplicated malaria cases risky. The best approach in these settings would be to proactively identify solutions to ACT stock-outs and maintain quinine as a fall-back drug only in case of ACT stock-outs. Additionally, improving quinine treatment outcomes by combining it with antibiotics, such as tetracycline or clindamycin [49-51], could be investigated and promoted. More recently, combinations of quinine and newer antibiotics with shorter treatment regimens that would improve adherence to therapy as well as minimize related adverse events have been evaluated. One such combination is that with azithromycin which is of particular interest, as the drugs act synergistically [52]. This combination offers promise for use especially in pregnant women and children < 8 years, since, unlike tetracyclines, both drugs are safe in these groups. A study in Thailand showed comparable efficacy in the treatment of multidrug resistant malaria, with cure rates of 100%, for a seven-day course of quinine+doxycycline and a three-day course of quinine+azithromycin [49]. These drug combinations will need further evaluation to confirm these findings and may offer a solution to the compliance problems associated with seven-day courses of quinine.

## Quinine for malaria in pregnancy

Malaria in pregnancy causes several adverse outcomes that include maternal anaemia, intrauterine growth retardation, low birth weight, preterm deliveries and abortion. Prevention and treatment of malaria in pregnancy is, therefore, critical to avoid these adverse outcomes. Currently the WHO recommends the use of quinine plus clindamycin for treating malaria in the first trimester of pregnancy, as the safety of artemisinin compounds during this period is not yet established [23]. As most clinical trials exclude women in their first trimester of pregnancy, information on the efficacy and safety of anti-malarial drugs during this period is extremely limited. Evidence for the safety of quinine in pregnancy is mostly historical and there are few clinical trials published [50,53]. Clindamycin on the other hand has a good safety record in pregnancy [54] and its pharmacokinetic properties are usually unchanged by pregnancy[55]. The combination of quinine and clindamycin has proven highly efficacious against multidrug-resistant strains of *P. falciparum*, with 42 day cure rates of 100% in one study [50]. The only concern with this combination is that it is usually not affordable for most resource limited settings. For the second and third trimester of pregnancy, quinine monotherapy seems to have unacceptably low efficacy in areas with multidrug resistant malaria when compared to ACT. Studies in these regions have shown that ACT performs better than oral quinine in terms of parasite clearance and fever clearance. Two studies in Thailand [56,57] reported fewer treatment failures at day 63 with artesunate plus atovaquone-proguanil and artesunate plus mefloquine, when compared with quinine. The occurrence of adverse events experienced by the pregnant women was similar in all groups, although tinnitus was more frequent in the quinine group. In these studies, the considerably inferior efficacy of quinine was attributed to both drug resistance and to the varying pharmacokinetic properties of quinine during pregnancy. In Africa however, available evidence suggests that *Plasmodium. falciparum *generally remains sensitive to quinine [58] and low cure rates with quinine monotherapy in pregnant women has been mainly attributed to poor compliance to treatment [59]. Thus in Africa, quinine monotherapy remains the most widely used treatment for malaria in the first trimester of pregnancy and is also considered safe during all trimesters of pregnancy. A recent study from Uganda provides important reassurance of continued efficacy of quinine monotherapy in these regions of Africa. In this study, quinine and artemether-lumefantrine had similar efficacy for the treatment of uncomplicated malaria in the second and third trimesters of pregnancy [60]. The evidence for safety of ACT use during the first trimester of pregnancy is currently limited [61]. Therefore, until more data become available, the recommendation to use quinine in the first trimester of pregnancy will remain and ACT should only be used in the second and third trimesters of pregnancy. Patient education and counseling will however be critical to promote compliance with therapy.

## Quinine in HIV or tuberculosis infected populations

Interactions between HIV and malaria remain a major public health concern in areas affected by both diseases. Very few studies have evaluated the role of quinine in the management of malaria in HIV infected populations. The earliest study was done in the Congo in 1986 and it showed malaria cure rates of 92% in HIV infected patients treated with oral quinine with comparable results in HIV-negative patients [62]. In a subsequent study in the same region, no significant differences in treatment response were observed between children with progressive HIV infection and HIV-uninfected controls treated with oral quinine [63]. Such findings and other available data suggest that malaria treatment policy in HIV infected populations can generally follow the standard practices. Concerns however remain about potential interactions between anti-malarial and anti-retroviral drugs. Currently, there is little published information on the co-administration of antiretroviral therapy (ART) and anti-malarial drugs, yet this will become increasingly important with the rapid scale-up of ART in Africa. In Nigeria, concurrent administration of nevirapine and quinine led to significant reductions in the plasma levels of quinine and elevated plasma levels of 3-hydroxyquinine, the major metabolite of quinine [64]. This could potentially reduce the efficacy of quinine while increasing toxicity, since 3-hydroxyquinine has higher toxicity and lower anti-malarial activity than quinine. Interactions with ritonavir have also been described, with concurrent administration of these drugs leading to marked elevations in plasma levels of quinine and decreases in levels of 3-hydroxyquinine [65]. These results suggest the need for downward dosage adjustments of quinine with concurrent administration of ritonavir, including ritonavir-boosted protease inhibitor regimens.

The co-existence of tuberculosis (TB), malaria and HIV in sub-Saharan Africa and other settings causes additional concerns about their treatment. Interactions between rifampicin (a major component of first-line anti-TB treatment regimens) and quinine would be expected as rifampicin is a potent inducer of hepatic enzymes and quinine is metabolised mainly by the human CYP 3A isoenzyme. In vivo studies in healthy volunteers showed that when quinine was administered with rifampicin its mean clearance was significantly greater and mean elimination half-life shorter [66]. Interesting observations of the effect of combined quinine and rifampicin therapy were additionally reported in Thai patients with uncomplicated malaria [67]. In this study, parasite clearance times were shorter in the quinine-rifampicin group than in the group given quinine monotherapy, suggesting that the anti-malarial activity of rifampicin augmented that of quinine initially. However, recrudescence rates were five times higher in the quinine-rifampicin group than in the quinine-alone group[67]. These observations were explained by marked differences in the plasma quinine concentrations when rifampicin was combined with quinine. These results suggest that the quinine dosage might need to be increased in patients receiving rifampicin as an anti-TB drug.

Concerns also exist about potential interactions with the concurrent use of antiretroviral drugs and artemisinin-based combination therapy [68-70]. Further research and pharmacovigilance will be critical to facilitate the development of targeted treatment recommendations. Presently, it is not possible to elucidate advantages associated with the use of any particular anti-malarial drug for HIV or TB infected populations.

## Quinine in the management of severe malaria

The treatment of severe malaria requires prompt, safe, and effective intravenous anti-malarial drugs. Over the years, quinine has been the mainstay in the treatment of severe malaria and still remains the first line drug in most African countries [24]. Though quinine dosing regimens have varied, the WHO recommends a dose of 20 mg salt/kg by intravenous infusion, then 10 mg/kg every eight hours [23]. The rationale for the loading dose is the urgent need to achieve therapeutic plasma concentrations. One systematic review showed that a loading dose of quinine reduced fever and parasite clearance times, but there was insufficient data to demonstrate its impact on risk of death [71].

More recently, intravenous artesunate is the recommended treatment of choice for severe falciparum malaria in adults [23]. This recommendation was made on the basis of the dramatic results of the SEAQUAMAT trial conducted in Southeast Asia that showed a 35% reduction in the case-fatality rate in adults with severe malaria treated with intravenous artesunate compared to intravenous quinine[20]. Subsequent systematic reviews have also provided additional evidence for this recommendation [72]. However, about 80% of malaria deaths occur in sub-Saharan Africa among children aged < 5 years. The therapeutic options previously recommended by WHO for the paediatric group included intravenous artesunate, intramuscular artemether or intravenous quinine[23]. Several trials and meta-analyses comparing intramuscular artemether with intravenous quinine have consistently shown no benefit of treatment with artemether over quinine in children with severe malaria in sub-Saharan Africa [73-75] (Table 2). The recently concluded AQUAMAT study now provides conclusive evidence of the superiority of intravenous artesunate over quinine in children <15 years, with a relative reduction of 23% in mortality associated with the use of artesunate[21]. These observations recently led to a change in WHO recommendations, with intravenous artesunate now advocated in preference to quinine for the management of severe malaria in children. The most critical issues that will need to be addressed, however, are the availability of intravenous artesunate for the patients who need it, especially in resource-limited settings, and its effectiveness in real-life settings. Until recently, the available formulations of injectable artesunate that have been used in several clinical trials were not produced according to Good Manufacturing Practices (GMP) and this could be a problem for African countries relying on donors who do not permit purchase of non-GMP artesunate. WHO recently pre-qualified intravenous artesunate manufactured by Guilin Pharmaceuticals in China and this may resolve problems of procurement of GMP artesunate. However, it is unclear whether supplies will be sufficient for the thousands of patients in need. Until these procurement and supplies issues are resolved, intravenous quinine may remain the only readily available drug for treating severe malaria in sub-Saharan Africa and other resource-limited settings. Furthermore, there are several health systems challenges related to the management of severe malaria in resource limited settings that impact on treatment outcomes, independent of the parenteral anti-malarial drugs used. Consequently, changes in treatment policies, in this case from quinine to artesunate, may not offer improvements without considering drug availability as well as additional measures to strengthen health systems.

**Table 2:** Summary of studies of quinine for the treatment of severe malaria

Study site	Year	Sample size and Study population	Drug Regimens	Treatment outcome	Comment	Reference
Gambia	1992-1994	576 children 1-9 years Cerebral malaria	Intramuscular artemether (IMA) Intravenous quinine (IVQ)	Mortality: IMA:20.5% IVQ: 21.5% Neurological sequelae: IMA: 3.3% IVQ: 5.3%	Artemether is as effective as quinine in treatment of cerebral malaria in children	[74]

Malawi	1992-1994	183 children Cerebral malaria	Intramuscular artemether (IMA) Intravenous quinine (IVQ)	Mortality: IMA: 11% IVQ: 16% Survival with neurological sequelae: IMA: 19% IVQ: 12%	Results do not suggest artemether would confer a survival advantage over quinine	[73]

Kenya	2000-2002	360 patients 1-60 years Severe malaria	IV Quinine + oral malarone (QM) IV Quinine +oral quinine (QQ)	Day 28 cure rates: QM: 98.7% QQ: 90%	Using malarone after IV quinine is safer and as effective as IV quinine +oral quinine	[87]

Burkina Faso	2001-2002	898 children 1-15 years Moderately severe malaria	Rectal quinine (RQ) Intramuscular quinine (IMQ)	Early treatment failure (day 3): RQ: 6% IMQ: 3% Fever recurrence on day 7: RQ: 5% IMQ: 10%	Rectal quinine had acceptable safety profile and could be used as early treatment for severe malaria	[84]

Uganda	2002-2003	103 children 0.5-5 years Cerebral malaria	Rectal artemether (RA) Intravenous quinine (IVQ)	Mortality: IVQ: 11.7% RA: 19.2%	Rectal artemether was effective and well tolerated	[76]

S.E Asia (Four countries)	2003-2005	1461 patients >2 years Severe malaria	Intravenous artesunate (IVA) Intravenous quinine (IVQ)	Mortality: IVA: 15% IVQ: 22% Absolute reduction in mortality: 34.7%	Intravenous artesunate should be treatment of choice for severe malaria in adults	[20]

Uganda	2003-2004	110 children 0.5-5 years Cerebral malaria	Rectal quinine (RQ) Intravenous quinine (IVQ)	Mortality: RQ: 7% IVQ: 9% Comparable clinical and parasitological outcomes	Rectal quinine was efficacious and could be used as a treatment alternative	[79]

Africa (Nine countries)	2005-2010	5425 children < 15 years Severe malaria	Intravenous artesunate (IVA) Intravenous quinine (IVQ)	Mortality: IVA: 8.5% IVQ: 10.9% Relative reduction in mortality: 22.5%	Parenteral artesunate should replace quinine as the treatment of choice for severe malaria	[21]

Another important aspect of severe malaria case management is pre-referral treatment, which is treatment given to a patient with severe malaria before they are referred to a health facility. This is critical, as most malaria deaths, especially in Africa, occur outside hospitals, either in the communities or at lower levels of care. Studies evaluating the role of rectal artesunate and artemether as pre-referral treatment have found these options to be highly efficacious [76,77]. However, the biggest challenge faced in resource limited settings has been the non-availability of these preparations in health facilities. A recent survey in Uganda found that rectal artemisinins were available in only 5% of the health facilities despite the fact that this is the recommended pre-referral drug [78]. A feasible alternative is rectal quinine, which has been found to have comparable efficacy with intravenous quinine in the management of severe malaria in children [79-84] (Table 2) and could play a more significant role than currently acknowledged as pre-referral treatment for severe malaria. More recent studies in Senegal and Mali provide additional support for the efficacy and feasibility of this route and also show that a pre-referral kit of rectal quinine was acceptable to both caretakers and health workers [85,86].

Following successful administration of parenteral treatment for severe malaria, it is recommended to continue with an oral anti-malarial drug once a patient is able to tolerate oral therapy. The current practice is to continue the same medicine orally as given parenterally to complete a full treatment course [23]. The options for oral continuation therapy that are available in many African settings would therefore include oral quinine or an ACT. In non-pregnant adults, doxycyline would also be added to either of these drugs and given twice daily for 7 days. Where available, clindamycin may be substituted in children, since doxycyline is contraindicated in this age group [23]. The choice of oral continuation therapy following initial parenteral treatment of severe malaria may also have an impact on clinical outcomes, particularly on parasite clearance, fever clearance and potentially the risk of recurrent parasitaemia. In this regard completing intravenous quinine treatment with an ACT instead of oral quinine may improve the overall treatment outcome of parenteral quinine therapy. Studies evaluating this approach to therapy are limited. A study in Kenya during 2000-2002, showed that completing the intravenous quinine dose with oral malarone (atovaquone + proguanil) was associated with improved clinical outcomes compared to intravenous quinine followed by oral quinine [87] (Table 2). Additional studies should explore other options, in particular ACT, for improving therapeutic outcomes with intravenous quinine treatment.

## Potential explanations for quinine treatment failure

### Quinine resistance

Parasite drug resistance is probably the greatest problem faced by malaria control programs worldwide and is an important public health concern. Over the years, malaria parasites have developed resistance to a number of commonly used anti-malarial drugs. However the development of resistance to quinine has been slow. Although its use started in the 17^th ^century, resistance to quinine was first reported in 1910 [88]. In comparison, resistance to chloroquine and proguanil emerged within only 12 [89] and 1 year [88,90] of their introduction, respectively. Resistance to quinine is usually low grade, with the drug retaining some activity but having its action delayed or diminished. Diminished sensitivity of *P. falciparum *to quinine has been widely documented in Asia [91] and South America [92] but it seems relatively uncommon in Africa where conflicting results of no resistance [93,94] or varying degrees of resistance [95], [96] have been reported. A recent study from Thailand showed significant reductions in efficacy of quinine, artemisinin and mefloquine when compared to previous reports from the same area, suggesting further increase in drug resistance in this region [97]. No convincing evidence of high grade quinine resistance in the treatment of severe malaria has been reported. Findings from a recent systematic review of about 435 clinical trials published between 1966 and 2002 showed that the recrudescence rates for quinine reported over these past 30 years remained roughly constant [98]. These findings are encouraging and may suggest that efficacy of quinine has been preserved.

### Variations in quinine pharmacokinetics

Treatment failures with quinine could also be explained by varying pharmacokinetic profiles of the drug. It is known that quinine pharmacokinetic properties and therapeutic responses vary with age, pregnancy, immunity and disease severity [99]. Also, as patients recover from malaria, there is usually an expansion of the volume of distribution and an increase in systemic clearance of quinine resulting in a decline in the average concentration of quinine in plasma [100]. These variations may lead to drug levels that may be inadequate to completely clear infection. The possibility that pharmacokinetic factors may explain quinine treatment failure was initially raised about 20 years ago when a Thai patient who had fatal severe malaria and apparent RIII resistance was found to have abnormally low levels of quinine despite adequate dosing [101]. Additional evidence for the impact of unusual quinine pharmacokinetics on treatment outcomes was provided by a more recent study describing early treatment failure in a patient with severe malaria with an abnormally high volume of distribution and increased quinine clearance, resulting in abnormally low quinine concentrations [102]. A few studies have proposed that an increase in the quinine dosage after the third day could compensate for declines in plasma drug levels during recovery, especially in areas with resistant *P. falciparum *[99]. However, this is not routinely practiced. Despite these anecdotal observations, there is little evidence for large variations in quinine pharmacokinetics [103] and the exact role that variations in drug levels play in quinine treatment responses is unclear.

### Quinine drug quality and treatment compliance

The quality of quinine used in routine care could play a key role in clinical outcomes. Poor quality drugs remain a problem worldwide and are a serious public health threat. A study in Nigeria evaluating the quality of different anti-malarial drugs found that 37% of 225 anti-malarial drugs did not meet the tolerance limits set by United Sates Pharmacopeia (USP) for the amount of active ingredient, and 46% of these were formulations of quinine [104]. In Congo, Burundi and Angola only 89% of the declared active substance was found in quinine tablets, with high quantities of impurities reported [105]. Another worrying situation was unveiled in a survey in Cameroon, where nearly 74% of 70 quinine samples had no active ingredient [106]. Several other studies have also described varying problems with quinine drug quality in different settings [107,108]. Ideally, branded anti-malarial drugs should be used, but unfortunately, branded quinine products are not universally available in Africa and other malaria endemic settings. In addition, national drug regulators need to strengthen their roles in the monitoring of anti-malarial drug quality.

Another potential explanation for quinine treatment failures may be poor compliance. Quinine's prolonged treatment course and significant tolerability problems may lead to poor compliance, and hence poor therapeutic outcomes [32,45,59]. In this aspect, ACT has an advantage over quinine since it is administered once or twice daily over three days. A recent study in Uganda showed comparable compliance on day 3 of treatment in patients taking either quinine or artemether-lumefantrine. However, non-compliance to quinine greatly increased with increasing days on therapy to about 44% by day 7[45]. Promotion of shorter courses of quinine, especially in combination with antibiotics, should improve compliance as well as treatment outcomes [39,109].

## Conclusion

In the near future, quinine will continue to play a significant role in the management of malaria, particularly in resource limited settings. Following the results of the SEAQUAMAT and AQUAMAT trials, artesunate is now recommended as the treatment of choice for severe malaria patients, with quinine only acting as an alternative when artesunate is not available. The role of rectal quinine as pre-referral treatment for severe malaria has not been fully explored, but this remains a promising intervention given the limited availability of rectal artemisinin preparations in resource limited settings. Quinine continues to play a critical role in the management of malaria in the first trimester of pregnancy, and will remain so until safer alternatives become available. The continued use of quinine in the management of uncomplicated malaria is a concern. Clearly, the seven day duration of therapy and thrice daily administration of quinine present a major challenge to completion of therapy, leading to sub-optimal treatment outcomes. In these situations, ACT is a better option given the simplicity of dosing and shorter treatment duration. However, because of the frequent ACT stock outs, the rapid withdrawal of quinine as a treatment option for uncomplicated malaria cases is risky. The best approach would be, besides improving the supply system, to maintain quinine as a fall-back drug in case of ACT stock-outs.

## Competing interests

The authors declare that they have no competing interests.

## Authors' contributions

AJ, PJR and UD conceived the idea and wrote the first draft of the manuscript. All authors read and approved the final version
